# Habitat Discontinuities Separate Genetically Divergent Populations of a Rocky Shore Marine Fish

**DOI:** 10.1371/journal.pone.0163052

**Published:** 2016-10-05

**Authors:** Enrique Blanco Gonzalez, Halvor Knutsen, Per Erik Jorde

**Affiliations:** 1 Center for Coastal Research, University of Agder, N–4604 Kristiansand, Norway; 2 Institute of Marine Research (IMR), Flødevigen, N–4817 His, Norway; 3 Centre for Ecological and Evolutionary Synthesis (CEES), Department of Biosciences, University of Oslo, P.O. Box 1066 Blindern, N–0316 Oslo, Norway; National Cheng Kung University, TAIWAN

## Abstract

Habitat fragmentation has been suggested to be responsible for major genetic differentiations in a range of marine organisms. In this study, we combined genetic data and environmental information to unravel the relative role of geography and habitat heterogeneity on patterns of genetic population structure of corkwing wrasse (*Symphodus melops*), a rocky shore species at the northern limit of its distribution range in Scandinavia. Our results revealed a major genetic break separating populations inhabiting the western and southern coasts of Norway. This genetic break coincides with the longest stretch of sand in the whole study area, suggesting habitat fragmentation as a major driver of genetic differentiation of this obligate rocky shore benthic fish in Scandinavia. The complex fjords systems extending along the western coast of Norway appeared responsible for further regional genetic structuring. Our findings indicate that habitat discontinuities may lead to significant genetic fragmentation over short geographical distances, even for marine species with a pelagic larval phase, as for this rocky shore fish.

## Introduction

Connectivity between geographically separated populations plays a pivotal role in populations dynamics and genetic diversity [1]. The presence of physical barriers [2], environmental clines [3] and anthropogenic disturbances [4] may prevent connectivity, while the lack of suitable conditions to satisfy the biological requirements of the species [5–6] will shape patterns of genetic structure. While obvious in some terrestrial and riverine systems, boundaries to connectivity can be more inconspicuous in the marine realm. The relatively large population sizes and high dispersal potential of marine organisms commonly result in lower intraspecific genetic differentiation compared to freshwater species [7]. However, some species display sharp genetic discontinuities or breaks due to a variety of historical and contemporary processes acting as barriers to dispersal and gene flow, such as patchiness of suitable habitat or discontinuities in the oceanographic regimes [8–10].

Biological attributes of species, such as dispersal ability and reproductive mode, also play an important role on population demography, connectivity and the location of genetic discontinuities or breaks [11–12]. In species with very limited dispersal potential, genetic breaks may arise and persist for many generations even in the absence of physical barriers to gene flow, whereas their occurrence in high gene flow species will only occur when a barrier to gene flow is present [13–14]. Populations living at the limit of their distribution range may also exhibit strong patterns of genetic structure due to lower individual fitness in relation to severe selection regimes favoring locally adapted genotypes [15–16]. Hence, species with low dispersal capabilities living in the limit of their distribution ranges may display strong signatures of genetic differentiation and low levels of genetic diversity associated to habitat fragmentation [13,17–18]. Integrating spatial information on habitat features with other ecological and genetic data can contribute to understand the mechanisms driving genetic variation among populations (see reviews [19–22]).

Corkwing wrasse (*Symphodus melops*) is a small rocky shore fish inhabiting coastal areas of the Northeast Atlantic and reaches its northern limit in Scandinavia. Previous population genetic studies on this species revealed a major genetic break across the North Sea, and ascribed the significant reduction in genetic diversity of the northern populations to the postglacial colonization of the Scandinavian Peninsula [2,23]. Significant phenotypic divergence in life-history traits between western and southern Norwegian populations [24] may indicate further isolation and subtle genetic divergence [25–26]. The current study aims at evaluate the relative role of geography and habitat heterogeneity on patterns of genetic population structure of corkwing wrasse, in an attempt to resolve underlying mechanisms behind cryptic genetic patterns in marine coastal species.

## Material and Methods

### The species and the study area

Corkwing wrasse (*Symphodus melops*) is a small benthic fish inhabiting the first few meters of the rocky shorelines of the Northeast Atlantic from southern Portugal to Norway and the western part of the Mediterranean Sea [27]. Since the second half of 1980’s, occurrence of corkwing wrasse in Scandinavia has increased greatly, in parallel with the water temperature increase registered in the area [2]. This species is now of commercial importance due to the high demand as cleaner fish by the salmon industry in northern Europe [28]. In contrast, its presence in the Mediterranean has declined and it is currently rare in the southern limit of its distribution range in Portugal [29]. Adult individuals show site tenacity [30], restricting species dispersal to the pelagic larval phase. Territorial males use seaweeds to build nests in rocky areas [31] and guard the benthic sticky eggs laid in the nest for 3–14 days (reviewed by Darwall et al. [32]). After hatching, pelagic larvae spend 3–4 weeks as part of the pelagic plankton prior to settlement (reviewed by Darwall et al. [32]).

**Fig 1:**
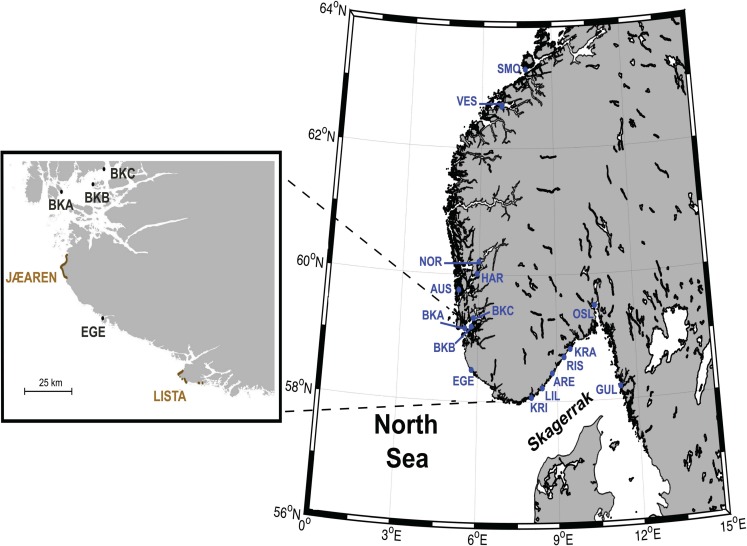
Sampling locations of corkwing wrasse analysed in this study (for details of sample abbreviations see Table 1). The location and geographical extension of the two major sandy areas in Norway, Jæren and Lista, are indicated in the inset.

The present study focuses on populations inhabiting the Norwegian coastline of North Europe. This area stands as the northern limit of distribution of many temperature species, including the corkwing wrasse, as well as the southern limit of some cold water species [33], with a trend towards warm water pelagic species being gradually more common in recent times [34]. The Norwegian coast is characterized by the predominance of rocky areas, except for an extensive sandy area along the coast of Jaeren and Lista covering 26 km between the southern tip of Norway and the southern limit of the deep western fjords (see Fig 1), and some very small sandy (“pocket”) beaches in coves [35], unsuitable for reproduction in corkwing wrasse (see review by Darwall *et al*. [31–32]). The fragmented coastline with a large number of fjords scattered along the coast and the disparities in the physico-chemical properties of the waters makes the Norwegian coast a complex system with the ideal conditions for retention of planktonic organisms, isolation of fjord populations, existence of sharp genetic breaks and, ultimately, vicariance [36–37].

### Sampling

The sampling design aimed at a geographically fine scale coverage of the southern and the western coast of Norway, to complement earlier investigations [2,23]. The sampling area extended from the Swedish east coast to slightly north of 63°N on the Norwegian west coast (cf. Fig 1). In total, we analysed 1437 fish collected at sixteen sampling localities, including eight localities from the western and eight localities from the southern Norwegian coast (details provided in Fig 1 and Table 1). Samples from southern localities were collected by a beach seine, while those from the west coast were collected by pots. Sampled fish were immediately transferred to a sea water tank and sacrificed in the most gentle and swift way by percussive stunning with a priest, in accordance with relevant legislation in Norway (Dyrevelferdsloven §12 (law of animal welfare: http://www.lovdata.no. Accessed 2016 July 30). Practices for sampling and handling of fish for this study were approved by the Norwegian Animal Research Authority and were performed by experienced personnel. The species is not protected by Norwegian law (it is a commercially harvested species in Norway), and no special permits were required either for research or commercially at sampled locations. Muscle tissue was taken from fresh or frozen specimens and stored in 96% ethanol prior to DNA extraction.

**Table 1:** Sample information and summary statistics for genetic variability in corkwing wrasse samples. n = sample size, *A* = average number of alleles per locus ± standard deviation, *A*r = average allelic richness per locus ± standard deviation. *H*_S_ = gene diversity. *F*_IS_ = deviation from Hardy-Weinberg genotype proportions. Numbers in bold indicate locus deviating significantly from Hardy-Weinberg expectation at 5% level after the False Discovery Rate approach [42].

Samplelocation	ID	Region	n	Latitude	Longitude	Year	A	A_r_	H_S_	F_IS_
Smøla	SMO	West	94	N 63.32	E 8.11	2015	12.1±7.3	11.0±6.5	0.687	0.008
Vestnes	VES	West	79	N 62.65	E 7.09	2011	11.9±7.6	11.0±6.7	0.667	0.008
Austevoll	AUS	West	71	N 60.09	E 5.26	2011	11.4±7.0	10.9±6.6	0.648	-0.007
Hardanger	HAR	West	72	N 60.12	E 5.91	2011	11.1±6.0	10.8±5.8	0.675	-0.003
Norheimsund	NOR	West	100	N 60.39	E 6.48	2014	12.2±7.2	10.9±6.6	0.679	-0.009
Bøknafjord A	BKA	West	96	N 59.21	E 5.50	2013	11.2±6.7	10.5±6.1	0.682	**0.018**
Bøknafjord B	BKB	West	95	N 59.25	E 5.78	2013	12.3±6.6	11.2±6.1	0.673	0.005
Bøknafjord C	BKA	West	96	N 59.34	E 5.88	2013	12.4±7.6	11.3±6.9	0.668	0.003
Egersund	EGE	Skagerrak	61	N 57.27	E 5.53	2008, 2009	7.5±4.5	7.5±4.5	0.574	0.003
Kristiansand	KRI	Skagerrak	96	N 58.11	E 8.34	2008, 2009, 2010	7.8±3.7	7.1±3.2	0.595	**0.055**
Lillesand	LIL	Skagerrak	87	N 58.20	E 8.27	2010	7.4±3.8	7.1±3.8	0.601	**0.089**
Arendal	ARE	Skagerrak	100	N 58.41	E 8.74	2014	8.2±3.7	7.3±3.5	0.557	0.039
Risør	RIS	Skagerrak	96	N 58.68	E 9.08	2010	7.8±4.3	7.2±3.9	0.590	-0.015
Kragerø	KRA	Skagerrak	96	N 58.88	E 9.38	2010	7.0±3.9	6.5±3.5	0.563	-0.007
Oslo	OSL	Skagerrak	99	N 59.52	E 10.39	2008, 2009	8.1±4.1	7.3±3.6	0.575	-0.005
Gulmarsfjord	GUL	Skagerrak	99	N 58.18	E 11.32	2008, 2009	8.1±4.0	7.6±3.8	0.562	0.004
Overall			1437						0.64	**0.002**

### Genetic analysis

Total genomic DNA was extracted from ethanol-preserved flesh using either the Viogene Inc. extraction kit (Sunnyvale, CA) or the DNeasy kit (Qiagen, Hilden, Germany), re-suspending the DNA in TE buffer. Microsatellite polymorphism was screened at nine polymorphic markers and following the same PCR protocols as described earlier for the species [2,38]. DNA fragments were run with GeneScan^Tm^-600 Liz as size standard on an ABI 3130XL automated sequencer. As a guard against potential genotyping errors, all samples were run with the same size standard and on the same machine as those previously used by Knutsen et al. [2]. Capillary traces where scored independently by two trained people, and disagreements were re-analysed (with new PCR of individuals) in order to avoid misclassification of alleles and genotypes.

### Statistical Analysis

Levels of genetic variation were characterized by counting observed alleles (*A*), allelic richness (*A*_r_) and gene diversity within samples (*H*_S_) and the total over all samples (*H*_T_), based on Nei & Chesser [39], using FSTAT software package (version 2.9.3.2; [40]). Deviations from Hardy-Weinberg (HW) equilibrium were investigated by the exact probability test in GENEPOP (version 4.0; [41]). Here, and in subsequent situations of multiple tests, we adopted the false discovery rate (FDR) approach [42] when interpreting the significance of individual tests within a larger table. Non-random association of alleles at different loci (i.e,”linkage disequilibrium”, LD) were tested in each sample separately by a G-test with 10 000 demorizations, 100 batches, and 1000 iterations per batch in GENEPOP (version 4.0; [41]). The presence of null alleles, stuttering errors or technical artifacts was investigated with MICROCHECKER (version 2.2.1; [43]).

Genetic differentiation among samples was quantified by Wright’s [44] *F*_ST_, using Weir & Cockerham’s [45] estimator *θ* applied to all samples, to pairs of sample localities and also within and among geographic regions. The statistical significance of observed genetic differentiation was estimated by 10 000 permutations in GENETIX (version 4.05; [46]). We adopted the FDR approach proposed by Benjamini & Yukutieli's [47] to correct for multiple tests in pairwise tables.

### Genetic Structure

Spatial patterns of genetic population structure were investigated using several approaches. First, we adopted the Bayesian clustering analysis implemented in STRUCTURE (version 2.3.3; [48]). This analysis was performed assuming an admixture model, running seven replicates for each value of *K* between 1 and 10 and with Markov chain Monte Carlo (MCMC) resampling using 100 000 repetitions after a burn-in of 100 000. The most likely number of clusters, *K*, was estimated as the value which maximized the averaged log-likelihood, log Pr(*X*|*K*) and the ad hoc statistic Δ*K* [49]. Once *K* was determined, individuals were assigned to the respective clusters and plotted with DISTRUCT (version 1.1; [50]).

Second, in order to visualize spatial patterns of population structure and locate discontinuities among corkwing wrasse populations, we used GENELAND [51] in R [52]. The analysis was run with 10 replicates setting the number of groups K between 1 and 10. MCMC resampling was set at 100 000 repetitions, 100 thinning and 200 burn-in period. The best result was chosen based on the highest average posterior probability. The analysis was performed under both the correlated and uncorrelated frequency models. The latter model may be over simplistic and miss the subtle patterns of population differentiation detectable with the correlated model; however, it may perform better under isolation-by-distance and prevent overestimation of K [51,53].

Third, a neighbour-joining (NJ) tree, based on the modified Cavalli-Sforza’s distance Da of Nei et al. [54], was constructed with the POPTREE software [55] to investigate the phylogenetic relationships between Norwegian populations of corkwing wrasse. The reliability of the tree topology was tested with 100 000 bootstrap replicates.

### Isolation-by-Distance and Isolation-by-Environment

We examined the relative importance of geographic distance (isolation-by-distance, IBD) and habitat discontinuity (isolation-by-environment, IBE), on spatial patterns of genetic differentiation (linearized pairwise *F*_ST_ estimates, *F*_ST_ ⁄ (1—*F*_ST_)). Pairwise geographic distances between sample locations were calculated following the coastline (range of distances between 12 and 1259 km). Patterns of IBD were investigated comparing genetic and geographic distance matrices by Mantel tests with 10 000 permutations in IBDWS [56]. For the IBE analysis, data on habitat type was downloaded from the Norwegian Environmental Agency (http://kart.naturbase.no/) and imported in QGIS [57]. The IBE analysis considered the effects associated with the presence of large sandy areas (i.e., absence of a rocky substrate), which are unsuitable habitats for reproduction in this species. The analysis was performed by dividing the study area into a grid of cells of 500 x 500 m and counting the number of cells of unsuitable habitats between each pair of sample locations. The presence of unsuitable sandy areas was only considered when a stretch of sand covered at least two consecutive cells of the grid. Two consecutive cells of unsuitable habitat were given a score or distance of 1 while suitable cells were given a value of 0. As isolation increases with the size of the habitat discontinuity, consecutive cells of unsuitable sand were assigned the square value of their counts, in order to account for the supposedly increased difficulty for the rocky shore species of transgressing larger sandy areas. Thus, localities separated by an area covering three consecutive cells of sand were assigned a distance of 4, while four consecutive cells was given a distance of 9, and so on (range of estimates between 0 and 70). The relative contribution of geographic and environmental distance to genetic differentiation was determined by partial Mantel test with 10 000 permutation in IBDWS [56] controlling for the effects of one factor at a time. The additive effects of geographic and environmental distance to genetic differentiation was further investigated following the multiple matrix regression with randomization (MMRR) approach proposed by Wang [58] with 10 000 permutations in R [52]. This approach was suggested to be especially robust under low to moderate gene flow. In this analysis, matrices were first standardized by subtracting the mean and dividing by the standard deviation.

## Results

### Summary statistics

Results were based on 1437 individuals genotyped at nine microsatellite markers with a successful coverage rate > 99% (only 54 missing genotypes, ranging between none and 10 individuals per locus). A total of 162 alleles were scored (S1 Table and S1 Fig). Levels of genetic variability in the pooled sample ranged among loci from a low for locus SMA107 (A = 7; *H*_T_ = 0.253) to high for SMB11 (*A* = 36; *H*_T_ = 0.832).

Overall, corkwing wrasse populations in this study were significantly structured at all loci (overall *F*_ST_ = 0.064, *P* < 0.001, S1 Table). Pairwise *F*_ST_ estimates averaged over loci ranged greatly among sample localities, from -0.002 to 0.150 (Table 2) with the largest differences found between western and southern Norwegian localities (overall *F*_ST_ = 0.107, *P* < 0.001, Table 3). Within the western and southern regions, the southern (Skagerrak) appeared genetically more homogeneous (overall *F*_ST_ = 0.003, *P* < 0.001) than did western samples (overall *F*_ST_ = 0.005, *P* < 0.001). Genetic differentiation within the western region appears to be driven largely by the two most northern samples (SMO and VES: cf. Table 2).

**Table 2:** Pairwise comparisons among corkwing wrasse sample localities within and across the three genetic breaks. Values below the diagonal are *F*_ST_ estimates for all sample pairs and above the diagonal are the corresponding *P*-values. Numbers in bold indicate statistical significant tests at 5% level after the False Discovery Rate approach [47].

	SMO	VES	AUS	HAR	NOR	BKA	BKB	BKC	EGE	KRI	LIL	ARE	RIS	KRA	OSL	GUL
SMO		0.000	0.000	0.019	0.000	0.000	0.000	0.000	0.000	0.000	0.000	0.000	0.000	0.000	0.000	0.000
VES	**0.0084**		0.000	0.000	0.000	0.000	0.000	0.000	0.000	0.000	0.000	0.000	0.000	0.000	0.000	0.000
AUS	**0.0086**	**0.0098**		0.372	0.163	0.120	0.011	0.064	0.000	0.000	0.000	0.000	0.000	0.000	0.000	0.000
HAR	0.0035	**0.0104**	0.0004		0.324	0.383	0.144	0.156	0.000	0.000	0.000	0.000	0.000	0.000	0.000	0.000
NOR	**0.0079**	**0.0108**	0.0015	0.0006		0.153	0.027	0.064	0.000	0.000	0.000	0.000	0.000	0.000	0.000	0.000
BKA	**0.0103**	**0.0107**	0.0020	0.0005	0.0015		0.943	0.985	0.000	0.000	0.000	0.000	0.000	0.000	0.000	0.000
BKB	**0.0136**	**0.0162**	0.0043	0.0016	0.0028	-0.0019		0.906	0.000	0.000	0.000	0.000	0.000	0.000	0.000	0.000
BKC	**0.0117**	**0.0128**	0.0023	0.0014	0.0020	-0.0022	-0.0016		0.000	0.000	0.000	0.000	0.000	0.000	0.000	0.000
EGE	**0.1046**	**0.1263**	**0.1127**	**0.0999**	**0.0983**	**0.0852**	**0.0853**	**0.0897**		0.050	0.118	0.520	0.460	0.267	0.033	0.817
KRI	**0.1024**	**0.1277**	**0.1141**	**0.0980**	**0.0983**	**0.0848**	**0.0816**	**0.0896**	0.0037		0.047	0.000	0.027	0.022	0.001	0.000
LIL	**0.0982**	**0.1233**	**0.1080**	**0.0944**	**0.0945**	**0.0817**	**0.0810**	**0.0862**	0.0026	0.0031		0.005	0.083	0.009	0.000	0.004
ARE	**0.1263**	**0.1495**	**0.1362**	**0.1204**	**0.1191**	**0.1039**	**0.1040**	**0.1107**	-0.0004	**0.0075**	0.0055		0.328	0.455	0.405	0.629
RIS	**0.1088**	**0.1296**	**0.1209**	**0.1055**	**0.1041**	**0.0902**	**0.0909**	**0.0967**	-0.0002	0.0034	0.0026	0.0004		0.883	0.160	0.413
KRA	**0.1229**	**0.1489**	**0.1369**	**0.1197**	**0.1190**	**0.1047**	**0.1049**	**0.1105**	0.0010	0.0038	0.0052	0.0000	-0.0018		0.554	0.472
OSL	**0.1179**	**0.1444**	**0.1344**	**0.1162**	**0.1169**	**0.1026**	**0.1029**	**0.1093**	0.0042	**0.0067**	**0.0079**	0.0000	0.0015	-0.0003		0.029
GUL	**0.1252**	**0.1481**	**0.1361**	**0.1216**	**0.1197**	**0.1040**	**0.1054**	**0.1098**	-0.0016	**0.0097**	0.0062	-0.0004	0.0004	0.0002	0.0036	

**Table 3:** Pairwise *F*_ST_ estimates within and among geographical regions.

	South	West	Europe*
South	0.003	0.107	0.163
West		0.005	0.076
Europe*			0.006

*) Values estimated using corkwing wrasse populations from the UK (data from [2]).

Genetic variability varied greatly among sample localities in the southern and the western regions (see Table 1 and S1 Fig for details). Briefly, western Norwegian samples consistently displayed higher levels of average genetic variability (averaged over samples and loci, *A*_r_ = 10.9; *H*_S_ = 0.672) compared to southern samples (*A*_r_ = 7.2; *H*_S_ = 0.577).

Deviations from Hardy-Weinberg (HW) expectations were significant in 11 of 144 (5.5%) cases. Four of them (2.8%) remained statistically significant at the 5% level also after FDR correction (Table 1: SMA11 in BKA, SMD112 and SMC8 in KRI and SMD112 in LIL), all due to a deficit of heterozygotes. MICROCHECKER suggested the presence of null alleles to explain the deficit of heterozygotes at locus SMC8 in KRI, but not in the other three cases. An examination of *F*_IS_ estimates for each allele separately in each sample did not indicate any pattern in the departure from HW genotype proportions (data not shown). Omitting one of the three locus at a time (SMA11, SMD112, SMC8) overall *F*_ST_ yielded similar results (i.e. *F*_ST_ changed from 0.064 to 0.063, 0.066 and 0.069; respectively); therefore, we decided to proceed the downstream analyses keeping all loci.

Non-random association of alleles at different loci (LD) were found to be significant (at the 5% level) in 30 of 576 pairwise tests (5.2%) before correction for multiple tests. Significant outcomes appeared randomly distributed among samples and only one pair (SMB11-SMD112 in SMO) remained statistically significant after the FDR approach (at the 5% level). Therefore, our results are consistent with the loci being independent.

### Pattern of genetic structure

**Fig 2:**
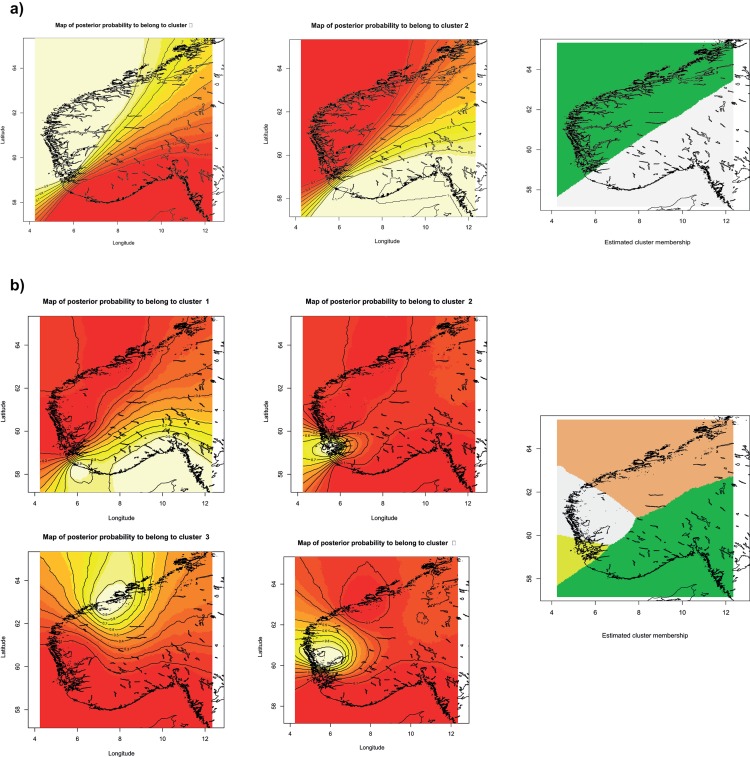
Maps of posterior probabilities of corkwing wrasse individual membership defined by GENELAND under a) uncorrelated (*K* = 2) and b) correlated (*K* = 4) alleles model. Plots representing assignment of pixels to each cluster (highest probability in light yellow and zero probability in red) and map of estimated posterior probability of population membership are presented for each model.

The STRUCTURE analysis uncovered two distinct clusters (K = 2) of sample localities (S2 Fig). One cluster included the samples collected from the southern coast while the other cluster corresponded to the samples from the western coast. Looking at further clustering scenarios, STRUCTURE suggested genetic admixture at samples KRI and LIL for K = 3. At K = 4, western samples showed significant genetic admixture and suggested an isolation-by-distance pattern between two clusters. Analysis assuming further clustering, i.e. K > 4, did not resolve further grouping of individuals. Under the uncorrelated allele method, GENELAND supported the existence of distinct western and southern groups (cf. Fig 2). On the other hand, the less conservative correlated method suggested four groups, with the west coast being divided in three groups, comprising a) those from Boknafjord (BKA, BKB and BKC), b) those collected around Hardangerfjord (AUS, HAR and NOR), and c) the two most northerly samples (SMO and VES).

**Fig 3:**
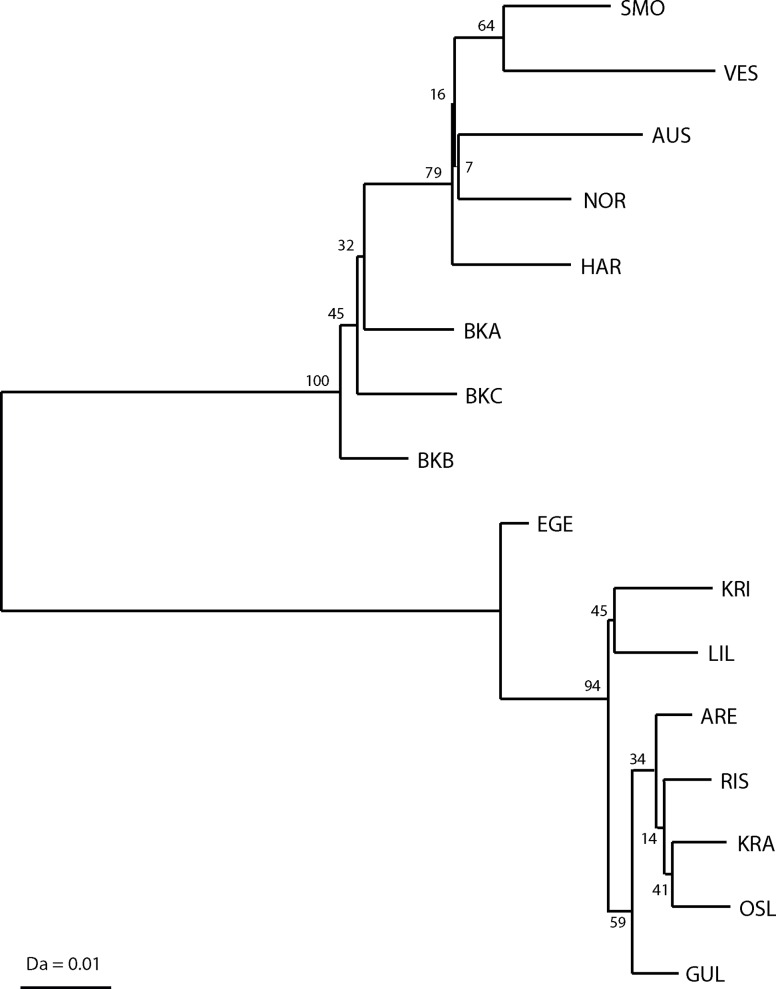
Unrooted neighbour joining tree based on Nei *et al*.’s [54] Da distances estimated for sixteen corkwing wrasse populations at nine microsatellite loci. Numbers of the nodes indicate the bootstrap support, based on 100 000 replicates.

The NJ tree based on Da genetic distance corroborated the existence of two major clusters (100% bootstrap support) comprising populations inhabiting the west coast of Scandinavia and the Skagerrak coast (Fig 3). The analysis suggested further subtle structuring within each region. In the west coast, the topology of the tree resembled the three groups suggested under the correlated alleles model by GENELAND_;_ i.e. Boknafjord, Hardangerfjord and northern samples (SMO and VES), with relatively high bootstrap support (64–79%). In the Skagerrak, EGE clustered apart from the rest of the samples (94% bootstrap support); nevertheless, branch lengths separating Skagerrak samples were usually shorter than those separating western Scandinavian samples.

### Isolation-by-Distance and Isolation-by-Environment

**Fig 4:**
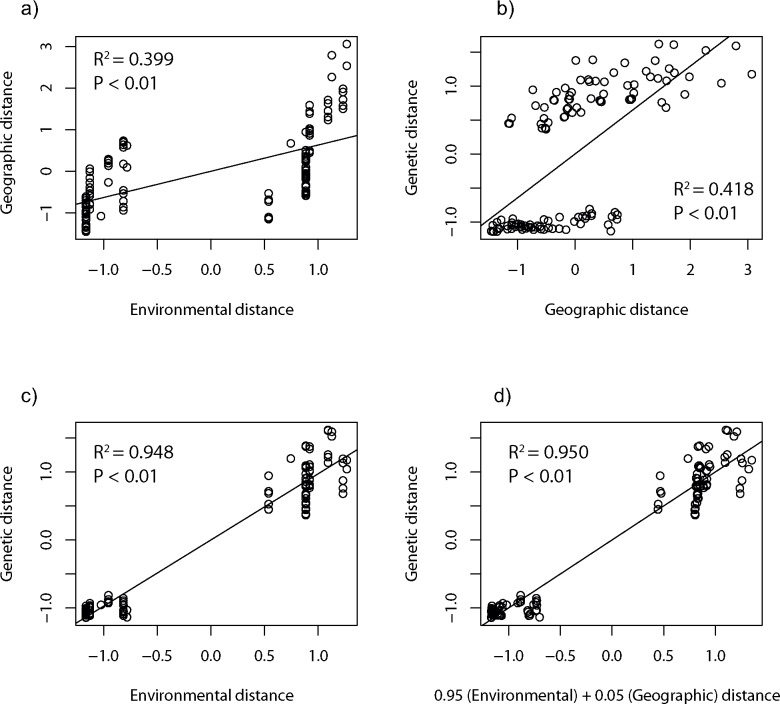
Results of multiple matrix regression with randomization (MMRR) analysis. Plots show a) the relationship of environmental and geographical distance, b) isolation-by-distance (IBD), c) isolation-by-environment (IBE), and d) multiple regression for the effects of geographical (ß_Distance_ = 0.05) and environmental (ß_Environment_ = 0.95) distances on genetic distance.

Matrices of standardized geographic and environmental (habitat type) distance showed moderate correlation (R^2^ = 0.399, *P* < 0.01, Fig 4A). Mantel tests of IBD showed significant correlation (R^2^ = 0.428, *P* < 0.01) between geographical distance and genetic differentiation, but gave considerably higher score to IBE as an explanatory variable for the spatial patterns of genetic structure (R^2^ = 0.938, *P* < 0.01). Genetic distance remained significantly correlated to both geographic distance (R^2^ = 0.049, *P* < 0.01) and environmental distance (R^2^ = 0.897, *P* < 0.01) when the influence of one of the other factor was controlled in partial Mantel tests (Table 4). Integrating genetic, geographic and environmental distance matrices under a multiple regression with randomization (MMRR) approach confirmed that environmental distance (IBE, ß_E_ = 0.95, *P* < 0.01) was a much stronger predictor of the observed genetic patterns than was geographic distance (IBD, ß_D_ = 0.05, *P* < 0.01) (Fig 4B–4D). The influence of the two factors (geographic and environment distance) was further investigated in order to understand regional patterns of genetic structure in each side of the genetic break, i.e. west and south Norway. In the southern coast, both partial Mantel tests and MMRR revealed that neither variable was correlated to genetic distance (Table 4). In the west coast, genetic differences showed significant but moderate correlation to geographic distance when controlling for environmental distances (R^2^ = 0.162, *P* = 0.02), whereas no correlation between genetic and environmental matrices was detected when controlling for geographic distances (R^2^ = 0.024, *P* = 0.25). The MMRR analysis confirmed that geographic distance (IBD, ß_D_ = 0.14, *P* < 0.01) was a stronger predictor of the observed genetic patterns than environmental distance (IBD, ß_E_ = 0.02, *P* < 0.01). Both factors together explained nearly 70% of the genetic variability in the west coast (Table 4).

**Table 4:** Results of the partial Mantel tests and MMRR analysis comparing the contribution of geographical (IBD) and environmental distance (IBE) to genetic differentiation for the whole Scandinavia, and separately for southern (Skagerrak) and West coast samples. r = coefficient of correlation, *P* = *P*-values (significant values in bold).

				Partial Mantel		MMRR		
Scandinavia			controlled by					
			Distance		Environment	Distance	Environment	Distance & Environment
	r		0.222		0.947	0.418	0.948	0.950
	*P*		**0.002**		**<0.001**	**<0.01**	**<0.01**	**<0.01**
Skagerrak								
	r		- 0.279		0.131	<0.001	0.062	0.077
	*P*		0.807		0.307	0.992	0.202	0.760
West								
	r		0.154		0.402	0.682	0.630	0.689
	*P*		0.250		**0.024**	**<0.01**	**<0.01**	**0.018**

## Discussion

The current study performed on corkwing wrasse collected along the Norwegian coast revealed the existence of a major genetic discontinuity or “break” in this rocky shore fish species. The genetic break was located in the south-western part of the Scandinavian Peninsula, separating populations inhabiting the western and southern (i.e., Skagerrak) coast of the Scandinavian Peninsula (*F*_ST_ = 0.107, *P* < 0.001, Table 3). This area is characterized by the presence of the longest stretch of sand (at Jæren) along the Norwegian coast.

Corkwing wrasse populations across the south-west Scandinavian genetic break displayed a marked difference in levels of genetic variability (both in number of alleles and heterozygosity), with higher levels in the west (cf. Table 1). These findings imply lack of gene flow across the break. It is possible that this marked genetic structuring of the species has its origin in processes, such as bottlenecks or founder effects, occurring during the (re)colonization of post-glacial Scandinavian waters, as discussed by Robalo et al. [23] and Knutsen et al. [2]. However, the fact that the genetic structure remains > 10 000 years later is a strong indication that whatever environmental feature(s) were responsible for generating it still operate to block gene flow in this species. Genetic differentiation across the Scandinavian Peninsula was of similar magnitude to that observed previously across the North Sea by Knutsen et al. [2] (cf. Table 3).

Our analyses suggested environmental distance in the form of long stretches of sandy areas to be a better predictor of current patterns of genetic population structure in Scandinavia than is geographical distance (Fig 4, Table 4). Long stretches of sandy area as a major obstacle to gene flow in corkwing wrasse is consistent with the reproductive mode of this obligate rocky shore species, e.g. nest building on rocky substrate [31–32] and low dispersal potential of adults [30]. Gene flow among western and Skagerrak populations could be possible during the few weeks that pelagic larvae travel as part of the pelagic plankton before settling to the bottom (reviewed by Darwall et al. [32]), but our results suggest no gene flow among populations across the putative south-west barrier (c.f. Fig 2 and S2 Fig). Selective forces may also lead to strong genetic differences, even in the presence of gene flow [59–60], but it seems highly unlikely that selection should operate on all investigated microsatellites to yield such an effect. Alternatively, western and southern populations could be adapted to different environmental conditions and therefore selected against, should they manage to cross the “barrier”.

Genetic breaks have usually been concordant with the location of strong historical oceanographic features and biogeographic breaks, reflecting the parallelism between the processes governing geographic and genealogical boundaries of the species [25,61–63]. Habitat discontinuity in relation to changes in bathymetry has been addressed to explain genetic breaks in a wide variety of rocky shore species [6,64–65]. However, examples of genetic breaks associated to the presence of sandy areas in rocky shore fish are scarce (but see [66–67]).

Regionally, corkwing wrasse revealed further patterns of genetic substructure (c.f. Table 2 and Fig 2 and S2 Fig). Western Scandinavian populations were genetically more differentiated than southern populations (Table 3). In the west coast, corkwing wrasse displayed a moderate isolation-by-distance pattern in genetic diversity (Table 4, Fig 4 and S2 Fig) with the presence of three major groups corresponding to samples around Boknafjord, Hardangerfjord and the two most northerly samples (SMO and VES) (Figs 2 and 3). Populations in the south were genetically fairly homogeneous (Tables 2 and 3) and the relatively strong coastal currents in the south [68] may favor gene flow among these southern localities. Results of the phylogenetric tree (Fig 3) clustered EGE apart from the rest of the Skagerrak samples (94% bootstrap support), and it is interesting to note that the second largest sandy area in Norway around Lista separate this locality from the other southern ones (see map for details, Fig 1). In contrast to the single stretch of sand in Jæren, sandy areas around Lista are interrupted by intermittent rocky areas which may facilitate some population connectivity. The presence of small fjords may be partly responsible for the subtle patterns of regional genetic population structure observed in corkwing wrasse as already reported for other coastal species in the Skagerrak [69–70].

Current findings have important implications for fisheries management and conservation, considering that corkwing wrasse is intensively exploited for use as a cleaner fish in the salmon industry [28,30]. A major concern lies in the fact that large numbers of individuals caught in the Skagerrak coast are translocated to salmon farms located in northern or western fjords, where temperature conditions are less suitable for the species [32]. Once salmons are harvested, net pens are emptied and wrasses released with no information of their fate, including putative hybridization with genetically different local stocks. Some of the concerns include the putative risk on the original genetic makeup of native stocks, differences in adaptive fitness or even extinction risk [71–72]. Hence, the strong genetic differences observed among western and Skagerrak populations suggest discontinuing present translocations among regions, and instead supplement salmon farms with local cleaner fish, as commonly recommended in stock enhancement programs [73].

## Supporting Information

S1 Fig Allele frequencies and size distributions among corkwing wrasse sampling localities at nine microsatellite markers. The size of the bubble corresponds to the frequency of the respective allele in the sample.
(PDF)

S2 Fig Results of the Bayesian clustering of corkwing wrasse from sixteen sample localities based on STRUCTURE. Each vertical bar in the left graph denotes an individual fish, whilst colours denote inferred clusters (*K* = 2 to 5). The right graph shows ΔK for different numbers of genetic clusters, suggesting K = 2 as the most likely outcome.
(PDF)

S1 Table Genetic variability at nine microsatellite loci. *A* = number of alleles, *H*T = gene diversity in the total material; *F*ST = estimate of *θ* (Weir & Cockerham 1984). Numbers in bold indicate statistical significant tests at 1% level after the False Discovery Rate approach (Benjamini & Hochberg 1995).
(PDF)

## References

[1] Cowen RK, Gawarkiewicz GG, Pineda J, Thorrold SR, Werner FE. Population connectivity in marine systems: an overview. Oceanography 2007;20: 14–21.

[2] Knutsen H, Jorde PE, Blanco Gonzalez E, Robalo JI, Albretsen J, Almada V. Climate change and genetic structure of leading edge and rear end populations in a northwards shifting marine fish species, the corkwing wrasse (*Symphodus melops*). PLoS One 2013;8: e67492 10.1371/journal.pone.0067492 23840721 PMC3694022

[3] Nanninga GB, Saenz-Agudelo P, Manica A, Berumen MI. Environmental gradients predict the genetic population structure of a coral reef fish in the Red Sea. Molecular Ecology 2014;23: 591–602. 10.1111/mec.12623 24320929

[4] Blanco Gonzalez E, Aritaki M, Knutsen H, Taniguchi N. Effects of large-scale releases on the genetic structure of red sea bream (*Pagrus major*, Temminck et Schlegel) populations in Japan. PLoS One 2015;10: e0125743 10.1371/journal.pone.0125743 25993089 PMC4439072

[5] Knutsen H, Jorde PE, Sannæs H, Rus Hoelzel A, Bergstad OA, Stefanni S, et al Bathymetric barriers promoting genetic structure in the deepwater demersal fish tusk (*Brosme brosme*). Molecular Ecology 2009;18: 3151–3162. 10.1111/j.1365-294X.2009.04253.x 19549108

[6] Kelly RP, Palumbi SR. Genetic structure among 50 species of the northeastern Pacific rocky intertidal community. PLoS One 2010;5: e8594 10.1371/journal.pone.0008594 20062807 PMC2799524

[7] DeWoody JA, Avise JC. Microsatellite variation in animal populations, with special emphasis on marine, freshwater, and anadromous fishes. J Fish Biol 2000;56: 461–473.

[8] Barton NH, Hewitt GM. Analysis of hybrid zones. Annual Review of Ecology and Systematics 1985;16: 113–148.

[9] Hellberg ME, Burton RS, Neigel JE, Palumbi SR. Genetic assessment of connectivity among marine populations. Bulletin of Marine Science 2002;70: 273–290.

[10] Ayre DJ, Minchinton TE, Perrin C. Does life history predict past and current connectivity for rocky intertidal invertebrates across a marine biogeographic barrier? Molecular Ecology 2009;18: 1887–1903. 19434808 10.1111/j.1365-294x.2009.04127.x

[11] Bradbury IR, Campana SE, Bentzen P. Estimating contemporary early life-history dispersal in an estuarine fish: integrating molecular and otolith elemental approaches. Molecular Ecology 2008;17: 1438–1450. 10.1111/j.1365-294X.2008.03694.x 18321254

[12] Sivasundar A, Palumbi SR. Life history, ecology and the biogeography of strong genetic breaks among 15 species of Pacific rockfish, *Sebastes*. Marine Biology 2010;157: 1433–1452.

[13] Irwin DE. Phylogeographic breaks without geographic barriers to gene flow. Evolution 2002;56: 2383–2394. 12583579 10.1111/j.0014-3820.2002.tb00164.x

[14] Haye PA, Segovia NI, Muñoz-Herrera NC, Gálvez FE, Martínez A, Meynard A, et al Phylogeographic structure in benthic marine invertebrates of the Southeast Pacific coast of Chile with differing dispersal potential. PLoS One 2014;9: e88613 10.1371/journal.pone.0088613 24586356 PMC3929388

[15] Johannesson K, André C. Life on the margin—genetic isolation and diversity loss in a peripheral marine ecosystem, the Baltic Sea. Molecular Ecology 2006;15: 2013–2029. 16780421 10.1111/j.1365-294X.2006.02919.x

[16] Orsini L, Vanoverbeke J, Swillen I, Mergeay J, Meester L. Drivers of population genetic differentiation in the wild: isolation by dispersal limitation, isolation by adaptation and isolation by colonization. Molecular Ecology 2013;22: 5983–5999. 10.1111/mec.12561 24128305

[17] Avise JC, Arnold J, Ball RM, Bermingham E, Lamb T, Neigel JE, et al Intraspecific phylogeography: the mitochondrial DNA bridge between genetics and systematics. Annual Review of Ecology and Systematics 1987;18: 489–522.

[18] Kawecki TJ. Adaptation to marginal habitats. Annual Review of Ecology, Evolution and Systematics 2008;39: 321–342.

[19] Galindo HM, Olson DB, Palumbi SR. Seascape genetics: a coupled oceanographic-genetic model predicts population structure of Caribbean corals. Current Biology 2006;16: 1622–1626. 16920623 10.1016/j.cub.2006.06.052

[20] Hansen MM, Hemmer-Hansen J. Landscape genetics goes to sea. Journal of Biology 2007;6: 6 18021427 10.1186/jbiol59 PMC2373898

[21] Selkoe KA, Henzler CM, Gaines SD. Seascape genetics and the spatial ecology of marine populations. Fish and Fisheries 2008;9: 363–77.

[22] Riginos C, Liggins L. Seascape genetics: populations, individuals, and genes marooned and adrift. Geography Compass 2013;7: 197–216.

[23] Robalo JI, Castilho R, Francisco SM, Almada F, Knutsen H, Jorde PE, et al Northern refugia and recent expansion in the North Sea: the case of the wrasse *Symphodus melops* (Linnaeus, 1758). Ecology and Evolution 2012;2: 153–164. 10.1002/ece3.77 22408733 PMC3297185

[24] Halvorsen KT, Sørdalen TK, Durif C, Knutsen H, Olsen EM, Skiftesvik AB, et al Male-biased sexual size dimorphism in the nest building corkwing wrasse (*Symphodus melops*): implications for a size regulated fishery. ICES Journal of Marine Science 10.1093/icesjms/fsw135

[25] Pelc RA, Warner RR, Gaines SD. Geographical patterns of genetic structure in marine species with contrasting life histories. Journal of Biogeography 2009;36: 1881–1890.

[26] Merilä J, Hendry AP. Climate change, adaptation, and phenotypic plasticity: The problem and the evidence. Evolutionary Applications 2014;7: 1–14. 10.1111/eva.12137 24454544 PMC3894893

[27] Quignard J-P, Pras A. Labridae In: Whitehead PJP, Bauchor M-L, Hureau J-C, Nielsen J, Tortonese E, editors. Fishes of the North-eastern Atlantic and the Mediterranean vol. II UNESCO, Paris, France 1986, pp. 919–942,

[28] Skiftesvik AB, Blom G, Agnalt A-L, Durif CMF, Browman HI, Bjelland RM, et al Wrasse (Labridae) as cleaner fish in salmonid aquaculture–The Hardanger fjord as a case study. Marine Biology Resources 2014;10: 289–300.

[29] Raventós N, Macpherson E. Planktonic larval duration and settlement marks on the otoliths of Mediterranean littoral fishes. Marine Biology 2001;138: 115–1120.

[30] Espeland SH, Nedreaas K, Mortensen S, Skiftesvik AB, Agnalt A-L, Durif CMF, et al. Kunnskapsstatus leppefisk–utfordringer i et økende fiskeri. Fisken og Havet 7/2010, 35 pages (in Norwegian).

[31] Uglem I, Rosenqvist G. Nest building and mating in relation to male size in corkwing wrasse, *Symphodus melops*. Environmental Biology of Fishes 2002;63: 17–25.

[32] Darwall WRY, Costello MJ, Donelly R, Lysaght S. Implications of life-history strategies for a new wrasse fishery. Journal of Fish Biology 2002;41 (Suppl B): 111–123.

[33] Perry AL, Low PJ, Ellis JR, Reynolds JD. Climate change and distribution shifts in marine fishes. Science 2005;308: 1912–1915. 15890845 10.1126/science.1111322

[34] Barceló C, Ciannelli L, Olsen EM, Johannesen T, Knutsen H. Eight decades of sampling reveal a contemporary novel fish assemblage in coastal nursery habitat. Global Change Biology 2016;22: 1155–1167. 10.1111/gcb.13047 26238690

[35] Corner GD. Atlantic coast and fjords In: The physical geography of Fennoscandia (ed Seppala M), pp. 203–228, Oxford University Press, NY, USA.

[36] Jolly MT, Jollivet D, Gentil F, Thiebaut E, Viard F. Sharp genetic break between Atlantic and English Channel populations of the polychaete *Pectinaria koreni*, along the north coast of France. Heredity 2005;94: 23–32. 15305173 10.1038/sj.hdy.6800543

[37] Olsen JL, Coyer JA, Stam WT, Moy FE, Christie H, Jorgensen NM. Eelgrass *Zostera marina* populations in northern Norwegian fjords are genetically isolated and diverse. Marine Ecology Progress Series 2013;486: 121–132.

[38] Knutsen H, Sannæs H. Development of twelve microsatellite loci in the corkwing wrasse (*Symphodus melops*). Conservation Genetic Resources 2009;1, 433–436.

[39] Nei M, Chesser RK. Estimation of fixation indices and gene diversities. Annals of Human Genetics 1983;47: 253–259. 6614868 10.1111/j.1469-1809.1983.tb00993.x

[40] Goudet J. FSTAT (Version 1.2): a computer program to calculate F-statistics. Journal of Heredity 1995;86: 485–486.

[41] Rousset F. GENEPOP’007: a complete re-implementation of the GENEPOP software for Windows and Linux. Molecular Ecology Resources 2008;8: 103–106. 10.1111/j.1471-8286.2007.01931.x 21585727

[42] Benjamini Y, Hochberg Y. Controlling the false discovery rate: a practical and powerful approach to multiple testing. Journal of the Royal Statistical Society B 1995;57: 289–300.

[43] Van Oosterhout C, Hutchinson WF, Wills DP, Shipley P. Microchecker: software for identifying and correcting genotyping errors in microsatellite data. Molecular Ecology Notes 2004;4: 535–538.

[44] Wright S. Isolation by distance. Genetics 1943;28:114–138. 17247074 10.1093/genetics/28.2.114 PMC1209196

[45] Weir BS, Cockerham CC. Estimating F-statistics for the analysis of population structure. Evolution 1984;38: 1358–1370. 28563791 10.1111/j.1558-5646.1984.tb05657.x

[46] Belkhir K, Borsa P, Goudet J, Chikhi L, Bonhomme F. GENETIX v.4.05 logiciel sous Windows pour la génétique des populations. Laboratoire Génome, Populations, Interactions CNRS UMR 5000, University of Montpellier II, Montpellier. 2004.

[47] Benjamini Y, Yekutili D. The control of the false discovery rate in multiple testing under dependency. The Annals of Statistics 2001;29: 1165–1188.

[48] Pritchard JK, Stephens M, Donnelly P. Inference of population structure using multilocus genotype data. Genetics 2000;155: 945–959. 10835412 10.1093/genetics/155.2.945 PMC1461096

[49] Evanno G, Regnaut S, Goudet J. Detecting the number of clusters of individuals using the software structure: a simulation study. Molecular Ecology 2005;14: 2611–2620. 15969739 10.1111/j.1365-294X.2005.02553.x

[50] Rosenberg NA. Distruct: a program for the graphical display of population structure. Molecular Ecology Notes 2004;4: 137–138.

[51] Guillot G, Mortier F, Estoup A. GENELAND: a computer package for landscape genetics. Molecular Ecology Notes 2005;5: 712–715.

[52] R Development Core Team. R: A Language and Environment for Statistical Computing. R Foundation for Statistical Computing, Vienna 2011.

[53] Guillot G. Inference of structure in subdivided populations at low levels of genetic differentiation—The correlated allele frequencies model revisited. Bioinformatics 2008;24: 2222–2228. 10.1093/bioinformatics/btn419 18710873

[54] Nei M, Tajima F, Tateno Y. Accuracy of estimated phylogenetic trees from molecular data. Journal of Molecular Evolution 1983;19: 153–170. 6571220 10.1007/BF02300753

[55] Takezaki N. POPTREE: Population tree construction. University of advanced studies. Hayama, Kanagawa, Japan. 2000.

[56] Jensen JL, Bohonak AJ, Kelley ST. Isolation by distance, web service. BMC Genetics 2005;6: 13 15760479 10.1186/1471-2156-6-13 PMC1079815

[57] QGIS Development Team. Quantum GIS Geographic Information System. Open Source Gespatial Foundation Project 2009 http://qgis.osgeo.org.

[58] Wang IJ. Examining the full effects of landscape heterogeneity on spatial genetic variation: a multiple matrix regression approach for quantifying geographic and ecological isolation. Evolution 2013;67: 3403–3411. 10.1111/evo.12134 24299396

[59] Limborg MT, Helyar SJ, de Bruyn M, Taylor MI, Nielsen EE, Ogden R, et al Environmental selection on transcriptome-derived SNPs in a high gene flow marine fish, the Atlantic herring (*Clupea harengus*). Molecular Ecology 2012;21: 3686–3703. 10.1111/j.1365-294X.2012.05639.x 22694661

[60] Sodeland M, Jorde PE, Lien S, Jentoft S, Berg PR, Grove H, et al ‘Islands of divergence’ in the Atlantic cod genome are projections of polymorphic chromosomal rearrangement. Genome Biology and Evolution 2016;8: 1012–1022. 10.1093/gbe/evw057 26983822 PMC4860689

[61] Kuo CH, Avise J. Phylogeographic breaks in low-dispersal species: the emergence of concordance across gene trees. Genetica 2005;124: 179–186. 16134331 10.1007/s10709-005-2095-y

[62] Teske PR, von der Heyden S, McQuaid CD, Barker NP. A review of marine phylogeography in southern Africa. South African Journal of Science 2011;107: 43–53.

[63] Toms JA, Compton JS, Smale M, von der Heyden S. Variation in palaeo-shorelines explains contemporary population genetic patterns of rocky shore species. Biology Letters 2014;10: 20140330 10.1098/rsbl.2014.0330 24966206 PMC4090554

[64] Hickey AJR, Lavery SD, Hannan DA, Baker CS, Clements KD. New Zealand triplefin fishes (family Tripterygiidae): contrasting population structure and mtDNA diversity within a marine species flock. Molecular Ecology 2009;18: 680–696. 10.1111/j.1365-294X.2008.04052.x 19215584

[65] von der Heyden S, Gildenhuys E, Bernardi G, Bowie RCK. Fine-scale biogeography: tidal elevation strongly affects population genetic structure and demographic history in intertidal fishes. Frontiers in Biogeography 2013;5: 29–38.

[66] Bernardi G. Barriers to gene flow in *Embiotoca jacksoni*, a marine fish lacking a pelagic larval stage. Evolution 2000;54: 227–234. 10.1554/0014-3820(2000)054[0226:BTGFIE]2.0.CO;2 10937199

[67] Riginos C, Nachman MW. Population subdivision in marine environments: the contributions of biogeography, geographical distance and discontinuous habitat to genetic differentiation in a blennioid fish, *Axoclinus nigricaudus*. Molecular Ecology 2001;10: 1439–1453. 11412367 10.1046/j.1365-294x.2001.01294.x

[68] Sætre R. The Norwegian coastal current—oceanography and climate Tapir Academic Press, Trondheim 159 pp; 2007.

[69] Jorde PE, Knutsen H, Stenseth NC. Population structuring of coastal cod (*Gadus morhua* L.) and the geographic extent of local populations. Marine Ecology Progress Series 2007;343: 229–237.

[70] Knutsen H, Jorde PE, Blanco Gonzalez E, Eigaard OR, Pereyra RT, Sannæs H, et al Does population genetic structure support present management regulations of the northern shrimp (*Pandalus borealis*) in Skagerrak and the North Sea? ICES Journal of Marine Science 2015;72: 863–871.

[71] Araki H, Schmid C. Is hatchery stocking a help or harm? Evidence, limitations and future directions in ecological and genetic surveys. Aquaculture 2010;308: S2–S11.

[72] Laikre L, Schwartz MK, Waples RS, Ryman N, The GeM Working Group. Compromising genetic diversity in the wild: unmonitored large-scale release of plants and animals. Trends in Ecology and Evolution 2010;25: 520–529. 10.1016/j.tree.2010.06.013 20688414

[73] Blanco Gonzalez E, Umino T. Managing the genetic resources in the intensive stock enhancement program carried out on black sea bream in Hiroshima Bay, Japan In: Caliskan M, editor. Analysis of genetic variation in animals. InTech, Rijeka, Croatia; 2011 pp. 217–230.

