# Supplementary material for: Habitat Discontinuities Separate Genetically Divergent Populations of a Rocky Shore Marine Fish
Source: PLoS One. 2016 Oct 5;11(10):e0163052. doi: 10.1371/journal.pone.0163052 (PMC5051803; doi:10.1371/journal.pone.0163052)

S1 Fig. Allele frequencies and size distributions among corkwing wrasse sampling localities at nine microsatellite markers. The size of the bubble corresponds to the frequency of the respective allele in the sample.

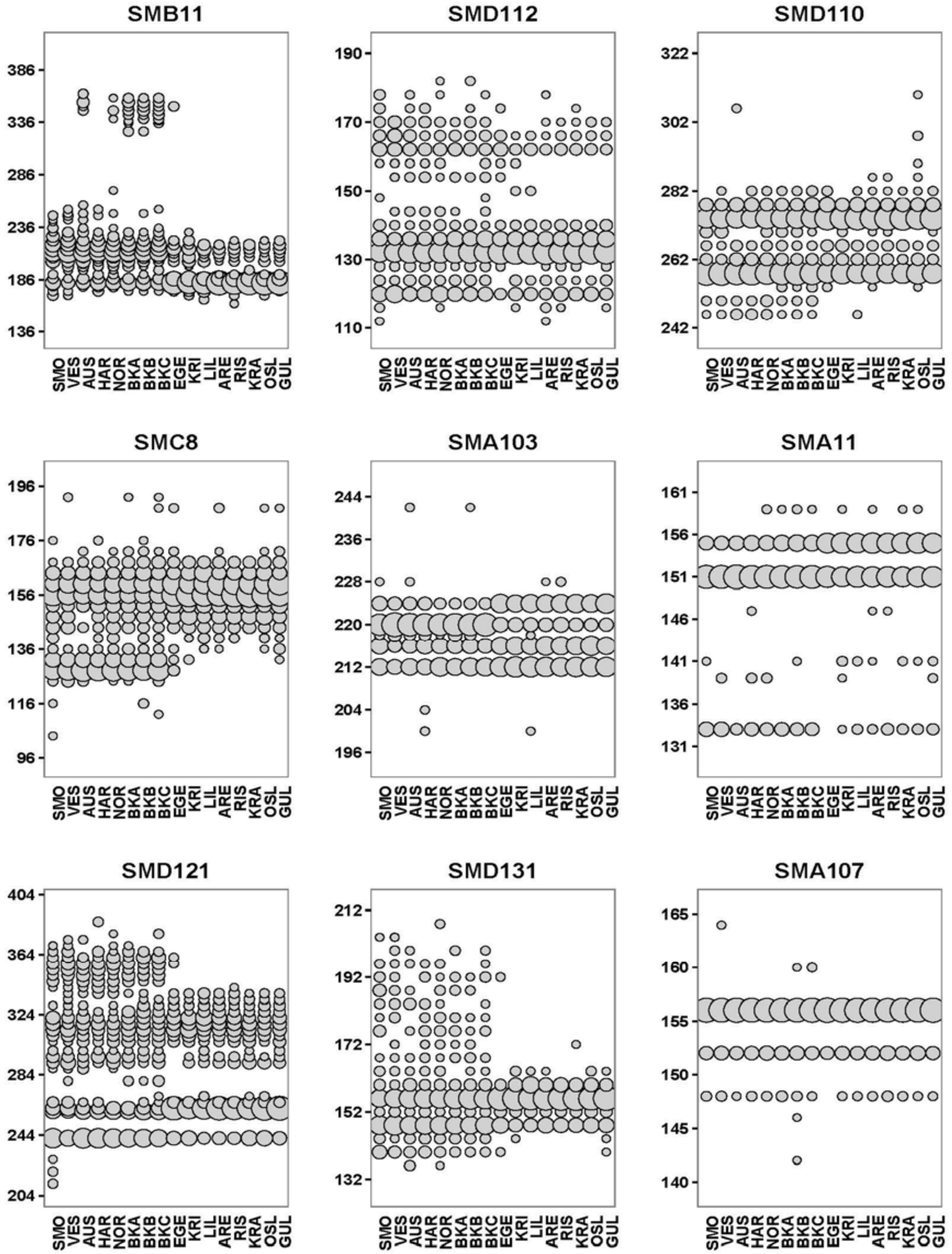

Supplement: S1 Fig — The size of the bubble corresponds to the frequency of the respective allele in the sample. (PDF) [file pone.0163052.s001.pdf]
