# Supplementary material for: Habitat Discontinuities Separate Genetically Divergent Populations of a Rocky Shore Marine Fish
Source: PLoS One. 2016 Oct 5;11(10):e0163052. doi: 10.1371/journal.pone.0163052 (PMC5051803; doi:10.1371/journal.pone.0163052)

S2 Fig. Results of the Bayesian clustering of corkwing wrasse from sixteen sample localities based on STRUCTURE. Each vertical bar in the left graph denotes an individual fish, whilst colours denote inferred clusters (*K* = 2 to 5). The right graph shows ΔK for different numbers of genetic clusters, suggesting K = 2 as the most likely outcome.

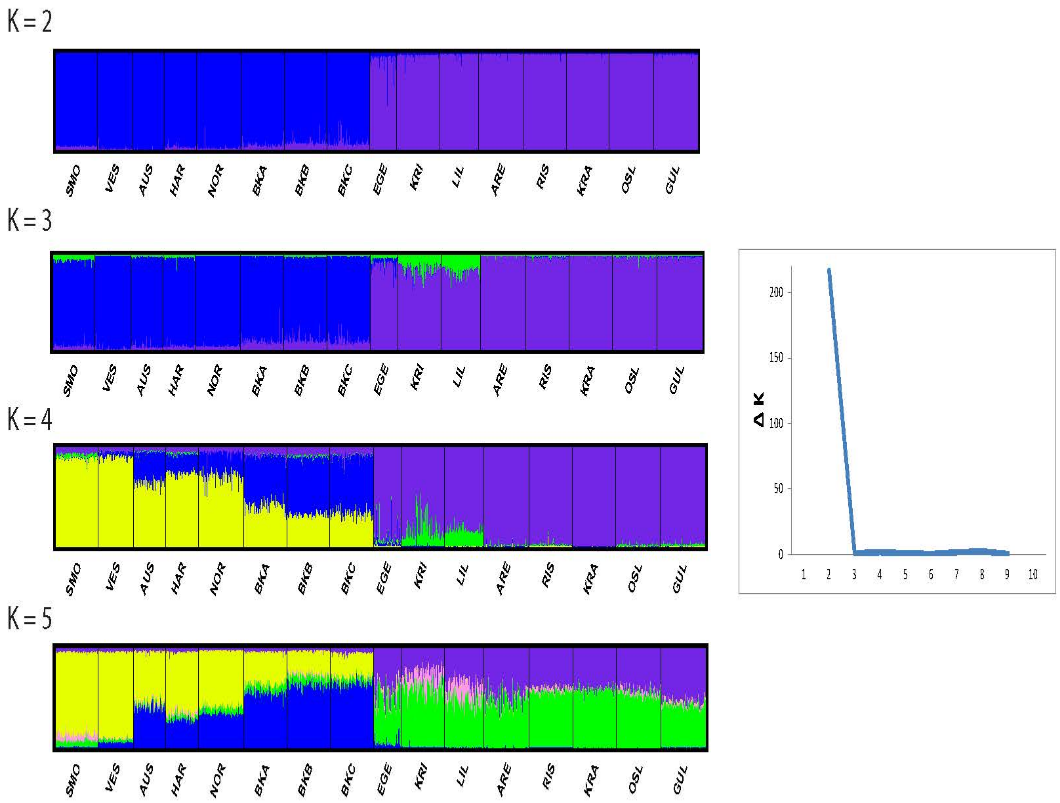

Supplement: S2 Fig — Each vertical bar in the left graph denotes an individual fish, whilst colours denote inferred clusters (K = 2 to 5). The right graph shows ΔK for different numbers of genetic clusters, suggesting K = 2 as the most likely outcome. (PDF) [file pone.0163052.s002.pdf]
