# Supplementary material for: Habitat Discontinuities Separate Genetically Divergent Populations of a Rocky Shore Marine Fish
Source: PLoS One. 2016 Oct 5;11(10):e0163052. doi: 10.1371/journal.pone.0163052 (PMC5051803; doi:10.1371/journal.pone.0163052)
Supplement: S1 Table — A = number of alleles, HT = gene diversity in the total material; FST = estimate of θ (Weir & Cockerham 1984). Numbers in bold indicate statistical significant tests at 1% level after the False Discovery Rate approach (Benjamini & Hochberg 1995). (PDF) [file pone.0163052.s003.pdf]

S1 Table. Genetic variability at nine microsatellite loci. A = number of alleles, H_T = gene diversity in the total material; F_{ST} = estimate of θ (Weir & Cockerham 1984). Numbers in bold indicate statistical significant tests at 1% level after the False Discovery Rate approach (Benjamini & Hochberg 1995).

Locus Name	A	H_T	F_{ST}
SMB11	36	0.794	**0.135**
SMD112	19	0.667	**0.018**
SMD110	15	0.659	**0.023**
SMC8	19	0.832	**0.049**
SMA103	9	0.745	**0.097**
SMA11	7	0.531	**0.069**
SMD121	33	0.877	**0.070**
SMD131	19	0.622	**0.059**
SMA107	7	0.253	-0.001
Average	18.2	0.665	**0.064**
SD	10.5		
